# The influence of teacher-student relationship on learning burnout in college students major in vocal music: a moderated mediation model

**DOI:** 10.3389/fpsyg.2026.1814971

**Published:** 2026-05-29

**Authors:** Yan Jiao, Siran Min

**Affiliations:** Vocal Teaching and Research Office, College of Music, Beihua University, Jilin, Jilin Province, China

**Keywords:** college students, learning burnout, perceived discrimination, self-efficacy, teacher-student relationship

## Abstract

To explore the mechanisms underlying learning burnout (LB) among college vocal music students, we tested a moderated mediation model involving the teacher-student relationship (TR), perceived discrimination (PD), and self-efficacy. Using cluster sampling, 729 students completed standardized questionnaires. Results from SPSS PROCESS macro revealed that TR negatively predicted LB, with PD partially mediating this effect. Furthermore, self-efficacy moderated this mediation, strengthening the protective effect of TR against PD for students with higher self-efficacy. These findings highlight targeted pathways to alleviate LB through enhancing TR and fostering self-efficacy.

## 1 Introduction

With the deepening advancement of China’s quality education strategy, the comprehensive development of students and aesthetic education have garnered increasing attention from both the state and society (Yang, 2020). Against this backdrop, universities across the country have expanded their enrollment scales, with arts programs—including vocal music majors—showing a marked trend of year-on-year growth in student intake (Huo and Jing, 2022). However, this expansion has been accompanied by increasingly prominent learning adaptation issues among arts students. Specifically, some students exhibit pronounced individualistic tendencies and narrow learning objectives; they place excessive emphasis on professional skill proficiency while neglecting non-specialized theoretical coursework; and they maintain overly emotional learning attitudes characterized by weak intrinsic motivation and a lack of persistence (Shou, 2010). Over time, these accumulated issues readily precipitate the phenomenon of “learning burnout”. Learning burnout (LB) refers to an extreme reaction students develop toward their studies when they are unable to effectively cope with academic pressure. It manifests as symptoms or behaviors such as low mood, negative attitudes, and inappropriate conduct. Students experiencing learning burnout lack interest or motivation in learning, feel bored and frustrated, and exhibit avoidance behaviors toward their studies (Paloș et al., 2019; Tuominen and Salmela, 2014). LB can lead to a series of maladaptive consequences, such as poor academic performance, truancy, dropping out of school, and may even result in psychological problems and disorders like anxiety and depression (Huebner et al., 2000; Rudolph et al., 2001). Therefore, exploring the influencing factors and internal mechanisms of LB in vocal music majors holds significant practical importance.

In higher education, college students majoring in vocal music face unique academic challenges, beyond conventional theoretical studies, they must endure intensive professional skill training, stage performance pressures, and fierce job competition (Liu, 2020). In the specialized one-on-one teaching model of vocal instruction, the quality of the teacher-student relationship (TR) significantly impacts the student’s psychological state (Gaunt, 2010). High-quality TR provide students with essential psychological safety and well-being (Concina, 2023), while poor power dynamics or emotional detachment readily trigger student anxiety, disillusionment, and even learning burnout (Carey and Grant, 2015). To delve deeper into the mechanisms underlying this phenomenon, this study aims to examine the impact of the TR on LB among college students majoring in vocal music. It introduces perceived discrimination as a mediating variable and investigates the moderating role of self-efficacy within this relationship.

While the available literature recognizes the important role of TR, a critical research gap exists. The specific psychological mechanisms by which PD and self-efficacy interact and associated with LB in the unique and high-pressure environment of vocal education have not been fully explored. Filling this gap is crucial for developing effective educational interventions. To explore the mechanisms behind this phenomenon more deeply, this study aims to investigate the impact of t TR on LB among college students major in vocal music.

## 2 Literature review and research hypotheses

### 2.1 The impact of teacher-student relationship on learning burnout

According to the Conservation of Resources Theory (COR), an individual’s psychological resources are finite. Prolonged exposure to environmental stressors leads to resource depletion, which in turn triggers extreme negative reactions such as burnout (Hobfoll, 1989). However, when individuals can obtain support from external environments to replenish their resources, this depletion process is effectively interrupted. Research indicates that external social support (such as high-quality TR) can serve as a crucial compensatory resource, helping students counteract negative perceptions within their environment and thereby significantly alleviate LB (Li and Zhang, 2024).

In the context of vocal music education at universities, the academic caliber and instructional approach of mentors and instructors directly related to students’ motivation to learn. Teachers’ attentive guidance and care can effectively reduce students’ feelings of burnout. Conversely, inadequate instruction or a distant TR can leave students feeling lost and demotivated. Compared to peer relationships, TR as vertical relationships, provide students with greater protection and security. Their predictive power regarding LB often surpasses that of peer relationships (Hartup, 1989; Verschueren and Koomen, 2012). In the unique context of higher music education, the one-on-one studio teaching model makes the teacher-student relationship highly professional and emotionally intensive (Miksza et al., 2020). Previous music education research has shown that this close interaction can provide both critical psychological support (Kuoppamäki et al., 2022) and a source of stress during power imbalances or lack of emotional resonance (Miksza et al., 2020; Ski-Berg, 2022).

Therefore, this study hypothesizes that TR as a protective factor, negatively related to LB among college students majoring in vocal music.

### 2.2 The mediating role of perceived discrimination

Although teacher-student relationships directly related to LB, their underlying mechanisms may operate through individuals’ cognitive evaluation processes. Perceived discrimination (PD) was a subjective experience reflecting the level at which individuals perceive unfair and negative treatment due to their group membership (Stevens and Thijs, 2018). Within educational ecosystems, PD acts as a covert and persistent external stressor, compelling students to expend substantial psychological resources to manage negative emotions and maintain self-worth. Empirical research confirms that heightened PD leads to greater resource depletion in countering this stress. When such internal consumption exceeds the rate of resource replenishment, students experience resource depletion, significantly elevating their risk of LB (Diamond et al., 2012; Hope et al., 2018).

According to the psychological mediation theory model, external environmental factors must be internalized into an individual’s psychological experience to exert a substantive predicted on their emotional expression (Fang, 2025). In daily teaching, positive TR can significantly satisfy students’ relational needs and enhance their sense of acceptance, thereby functioning as a psychological buffer to markedly reduce individuals’ perception of external discrimination (Wu and Gong, 2026). Conversely, distant or conflictual teacher-student interactions disrupt students’ social connections, intensify their subjective experience of isolation, and subsequently trigger defensive perceptions of environmental hostility and heightened discrimination awareness (Longobardi et al., 2026). This energy-draining negative psychological perception ultimately elevates the risk of severe LB. For university college students majoring in vocal music, perceiving neglect or unfairness from teachers—authoritative evaluators—can trigger self-doubt, leading to avoidance and LB. Therefore, this study hypothesizes that PD mediates the TR and LB among college students majoring in vocal music.

### 2.3 The moderating role of self-efficacy

According to the individual-environment interaction model, the buffering effect of external social support against negative environmental stress was not uniform but profoundly influenced by an individua’s internal psychological traits (Lyons et al., 2017). As a core component of psychological capital, self-efficacy determines whether an individual can effectively mobilize and transform external resources when facing potential threats (Bandura, 2015). Empirical research indicates that individuals with high self-efficacy possess greater confidence and a stronger sense of control, they can more efficiently transform external support into a psychological shield against hostile external influences, thereby significantly amplifying the mitigating effect of external support on perceived stigma (Wang et al., 2022). Based on this, the present study hypothesizes that while the TR can reduce LB, this effect varies depending on an individual’s level of self-efficacy.

According to social cognitive theory, self-efficacy refers to an individual’s belief and confidence in their ability to successfully cope with and accomplish specific tasks (Bandura et al., 1999). When confronting external pressures, self-efficacy serves not only as an indispensable internal psychological resource but also plays a crucial mediating role in the environment-cognition interaction (Xanthopoulou et al., 2007). Research indicates that individuals’ efficacy beliefs profoundly alter their cognitive evaluation mechanisms regarding external environmental factors (Bandura, 1999). Even when confronted with identical external environmental inputs, individuals possessing high self-efficacy resources can more effectively transform environmental support and reduce sensitivity to negative cues, thereby significantly moderating the ultimate impact of environmental factors on individual cognition. Recent studies increasingly highlight the dynamic role of self-efficacy in complex academic contexts. For instance, self-efficacy is closely linked to diverse learning approaches and technological acceptance in academic settings (Sun et al., 2025a, b), and it serves as a vital psychological resource in mitigating academic anxiety and enhancing learning enjoyment (Sun et al., 2026). Existing research also indicates that self-efficacy was significantly negatively correlated with PD and, as a protective resource, moderates the relationship between stress and adverse outcomes (Wang et al., 2025). Research on undergraduate music education students indicated that academic self-efficacy mediated the relationship between professional identity and LB. Enhancing music students’ self-efficacy not only directly reduces feelings of LB but also serves as a powerful internal resilience resource, internalizing and amplifying the positive impacts of the external environment (Zhu, 2025). Therefore, incorporating self-efficacy into the research model helps clarify under what conditions the teacher-student relationship can more effectively alleviate LB by reducing PD. Thus, this study hypothesizes that self-efficacy moderates the relationship in the model: “Teacher-Student Relationship → Perceived Discrimination → Learning Burnout.”

### 2.4 The present research hypotheses

**Figure 1:**
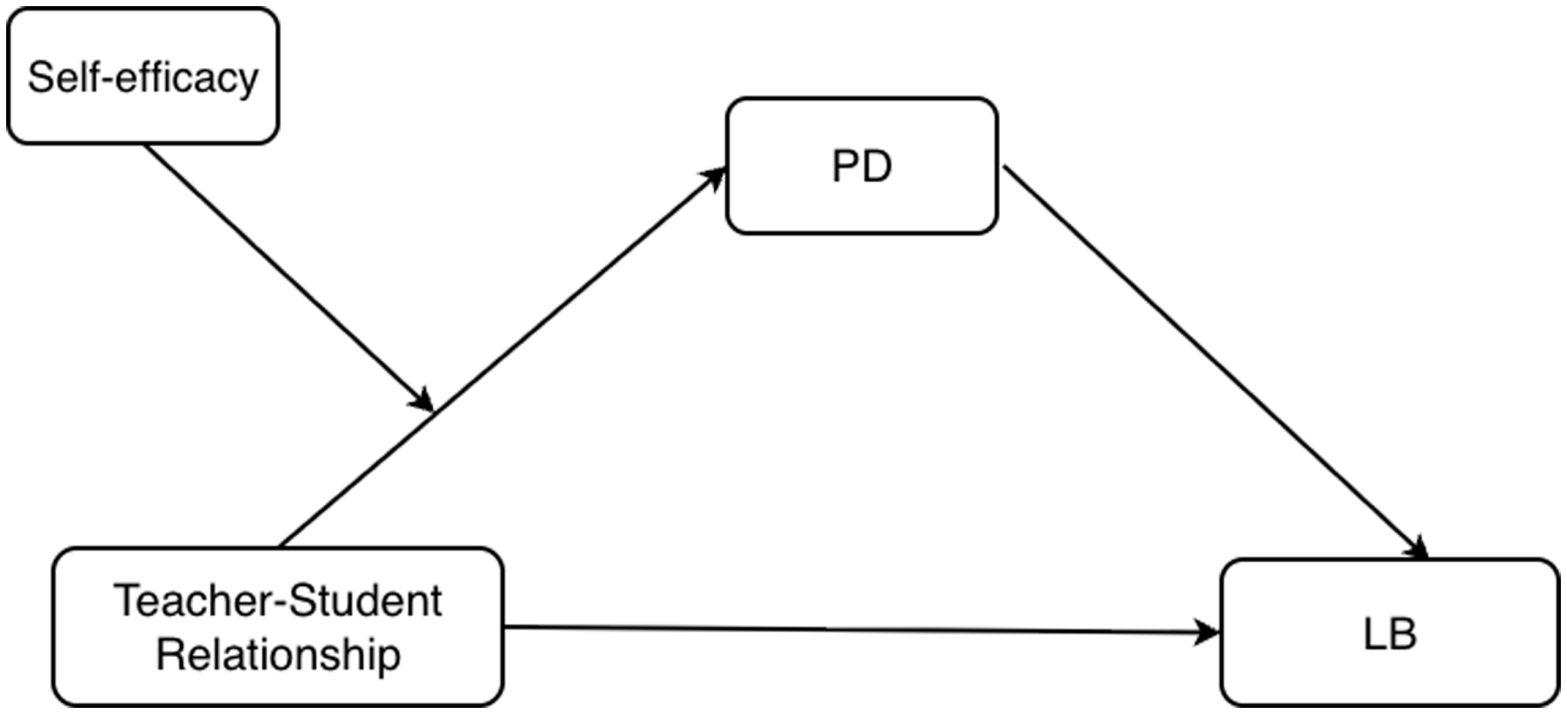
Hypothesized model.

This study explores the psychological mechanisms underlying low academic achievement among Chinese college students majoring in vocal music, aiming to develop targeted interventions to enhance their academic performance and mental health. To this end, we constructed a moderated mediation model with the teacher-student relationship as the independent variable and LB as the dependent variable (see Figure 1). Specifically, we hypotheses that:

*H1*: Higher TR will predict lower LB indirectly through reduced PD;

*H2*: The protective effect of TR against PD would be stronger for students with high self-efficacy.

## 3 Materials and methods

### 3.1 Participants and procedure

Using the cluster sampling method, 803 vocal music freshmen to senior students from three universities in Jilin Province, China, were selected as research objects. The inclusion criteria for sampling required participants to be full-time undergraduate students officially enrolled in a vocal music major, with active attendance during the survey period. Students who were on long-term medical leave, participating in off-campus internships, or declined to provide informed consent were excluded. 74 valid questionnaires were excluded, and a total of 729 valid questionnaires were obtained. The effective recovery rate was 90.78%. The mean age of the participants was 20.96 years (standard deviation 1.27, age range 18 to 23 years), of which 363 (48.8%) were male and 366 (49.2%) were female.

First, the Ethical Review Committee of Beihua University approved the study with approval number BHU-2025132. Second, we obtained informed consent from the participants. The students participated in the study anonymously. Third, our research was helped by teachers. In vocal classes, we distribute and recycle electronic questionnaires through the Questionnaire Star web platform with the help of teachers. Fourth, the graduate student in professional psychology is responsible for the survey and is responsible for interpreting the explanation of the questions asked by the participants.

### 3.2 Measures

For all Measurement questionnaire, a rigorous forward-and-backward translation process was conducted to ensure semantic equivalence. Furthermore, minor wording adaptations were made by a panel of psychology experts and vocal music educators to align the items with the specific educational context of Chinese vocal music students (e.g., contextualize “classmates” in QPD as “professional peers”). At the suggestion of the reviewers, we used Mplus 8.3 to test the structure validity of the questionnaire, and the Confirmatory Factor Analysis (CFA) results indicated that the scale had good construct validity in this study sample, *χ* ^2^/df = 2.45, CFI = 0.94, TLI = 0.93, RMSEA = 0.06.

#### 3.2.1 Individual parent-peer attachment questionnaire (IPPA)

A simplified version of the Individual Parent-Peer Attachment questionnaire was adopted (IPPA) (Armsden and Greenberg, 1987; Raja et al., 1992) to measure the attachment relationship between college students and teachers. The questionnaire consists of 10 items, with 3 items each in the trust and communication dimensions, such as “he/she respects my feelings” and “he/she will ask me if something bothers me”, and 4 items in the alienation dimension, such as “Discussing my problems with (the teacher) makes me feel ashamed or stupid”. This study only examines the attachment relationship reported by college students with teachers. It employs a five-point Likert scale, where 1 indicates “completely disagree” and 5 indicates “completely agree.” The overall attachment score was calculated based on the subjects’ scores on the three subscales (reverse scoring of the alienation dimension). The questionnaire Cronbach’s *α* = 0.95 indicates that the items are highly consistent and can reliably capture students’ attachment to the teacher.

#### 3.2.2 Learning burnout scale (LBS)

Learning burnout was measured using the Learning Burnout Scale (LBS) (Hu and Dai, 2007). The scale comprises 14 items covering four dimensions: academic inefficacy (e.g., “I feel incapable of achieving good grades”), emotional distress (e.g., “I’ve recently felt mentally lost, unsure of what to do”), physical exhaustion (e.g., “Lately, I often feel utterly drained and uninterested in anything”), and academic disengagement (e.g., “I want to give up studying”). It employs a five-point Likert scale, where 1 indicates “completely disagree” and 5 indicates “completely agree.” Higher scores indicate greater levels of LB. In this study, Cronbach’s *α* = 0.95, indicating the scale reliably captures students’ experiences of LB in terms of learning helplessness, emotional exhaustion, and negative learning attitudes.

#### 3.2.3 Perceived discrimination questionnaire (QPD)

Perceived discrimination was measured used the Perceived Discrimination Questionnaire (QPD) (Shen et al., 2009). The questionnaire comprises 15 items across four dimensions: intellectual and academic discrimination (e.g., “My classmates think I’m stupid and don’t want to play with me”), physical trait discrimination (e.g., “Classmates make fun of my unfashionable clothes”), language communication discrimination (e.g., “Classmates mock my unclear speech”), and family background discrimination (e.g., “I feel some classmates ridicule my parents’ jobs”). It employs a five-point Likert scale, where 1 indicates “completely disagree” and 5 indicates “completely agree.” Higher scores indicate greater perceived discrimination. The scale demonstrated Cronbach’s *α* = 0.93, reliably capturing students’ perceived discrimination pressure in school settings.

#### 3.2.4 General self-efficacy scale (GSES)

This study employed the General Self-Efficacy Scale (GSES) to measure self-efficacy, comprising 10 items such as “I am confident that I can effectively handle any unexpected events” (Schwarzer et al., 1999). It employs a 5-point Likert scale, where 1 indicates “strongly disagree” and 5 indicates “strongly agree”; higher scores reflect greater levels of general self-efficacy. In this study, the scale demonstrated excellent internal consistency with Cronbach’s α = 0.93.

### 3.3 Data analysis

First, we employed Harman’s single-factor test to assess common method bias (CMB) across all scale items (including four questionnaires covering the teacher-student relationship, learning burnout, self-efficacy, and perceived discrimination, totaling 49 items) (Podsakoff et al., 2003). The results indicated that this single factor explained only 31% of the total variance, well below the 40% threshold criterion. This suggests that common method bias was not significant in this study, meaning respondents’ subjective reports were not severely distorted by systematic bias.

Second, descriptive statistics and correlation analyses were conducted on the variable data using SPSS 26.0. Third, we employed the SPSS PROCESS macro 4.1 (Hayes, 2014) (Models 4 and 7) to examine the mediation model and the moderated mediation model. Controlling for age and gender variables, we tested the mediation model using Bootstrap analysis. All continuous variables were standardized in data analysis, and the interaction terms were computed using these standardized scores. The bootstrap method based on 5,000 samples was used to obtain the bias-corrected and accelerated 95% confidence intervals (CI) for the moderated mediation model. Effects were considered significant if the CI did not include zero. Finally, we conducted simple slope tests to interpret the magnitude of the moderation effect.

## 4 Results

### 4.1 Descriptive statistics

The results of descriptive statistics and correlation analysis are presented in Table 1. All variables were significantly correlated with each other (*p* < 0.01). Specifically, TR showed significant negative correlations with LB (r = −0.18) and PD (r = −0.17). PD was positively correlated with LB (r = 0.63) and negatively correlated with self-efficacy (r = −0.09). Self-efficacy was negatively correlated with LB (r = −0.22) and positively correlated with TR (r = 0.49). Our findings indicate that TR, PD, and self-efficacy are directly related to LB and collectively associated with LB.

**Table 1:** Descriptive statistics and correlation among variables (*N* = 729, Bootstrap = 5,000).

Predictors	*M*	*SD*	1	2	3	4	5	6
1 Age	20.96	1.27	1					
2 Gender	1.5	0.5	−0.16	1				
3 TR	3.93	0.82	−0.02	0.01	1			
4 PD	1.99	0.95	−0.08*	−0.12**	−0.17**	1		
5 Self-efficacy	3.65	0.84	−0.03	−0.07	0.49**	−0.09*	1	
6 LB	2.08	0.93	−0.05	−0.08*	−0.18**	0.63**	−0.22**	1

TR = teacher-student relationship; PD = perceived discrimination; LB = learning burnout; ^*^p < 0.05, ^**^p < 0.01.

### 4.2 Test for mediating effect

First, we employed Model 4 from the Process plugin to examine the mediating effect of PD between the teacher-student relationship and LB (see Table 2). Teacher-student attachment exerted a significant negative related to learning burnout among art students, with a total effect coefficient of −0.21 (*p* < 0.001), confirmed Hypothesis 1(H1).

**Table 2:** Test for mediating effect (*N* = 729, Bootstrap = 5,000).

Predictors	Model 1 LB		Model 2 PD		Model 3 LB	
	*β*	*t*	*β*	*t*	*β*	*t*
TR	−0.18	−4.95^**^	−0.17	−4.51^**^	−0.08	−2.72^**^
PD					0.62	21.33^**^
*R^2^*	0.03		0.03		0.41	
*F*	24.53^**^		20.25^**^		247.42^**^	

TR = teacher-student relationship; PD = perceived discrimination; LB = learning burnout; ^**^p < 0.01.

Further tests revealed (see Table 3) that perceived discrimination partially mediated this causal pathway: the indirect effect a*b was −0.12, Bootstrap 95% confidence interval [−0.18, −0.06] (excluding zero), accounting for 57% of the total effect.

**Table 3:** Test of the mediation effect between TR and LB.

Type	*β*	*SE*	95% CI	REV
Indirect effect	−0.12	0.03	[−0.18, -0.06]	57%
Direct effect	−0.09	0.03	[−0.12, -0.04]	43%
Total effect	−0.21	0.03	[−0.24, -0.13]	100%

Total effect, direct effect and indirect effect (*N* = 729, Bootstrap = 5,000). SE = standard error; CI = confidence interval; REV = relative effect value.

### 4.3 Test for moderated mediation

To further explore the potential moderating conditions under which TR predicted LB among college students major in vocal music, this study constructed a mediation model with moderation. It examined the moderating role of self-efficacy in the relationship between TR and LB via PD. The results are presented in Table 4, with detailed analysis as follows.

**Table 4:** Testing for moderated mediation (*N* = 729, Bootstrap = 5,000).

Predictors	PD			LB		
	β	SE	t	β	SE	t
Age	−0.05	0.03	−1.99*	−0.05	0.02	−0.25
Gender	−0.26	0.07	−3.77**	−0.06	0.05	−0.11
TR	−0.21	0.05	−4.25***	−0.09	0.03	−2.72**
PD				0.61	0.03	21.03***
self-efficacy	−0.02	0.05	−0.47			
TR* self-efficacy	−0.11	0.04	−3.01**			
R2	0.06			0.41		
F	9.31***			123.39***		

TR = teacher-student relationship; PD = perceived discrimination; LB = learning burnout; **p < 0.01, ***p < 0.001.

As proposed in Hypothesis 2, self-efficacy moderates the relationship between TR and LB through PD. Model 7 in the Process plugin (the first half-path in the moderation-moderation model) was used to test the moderation effect. Controlling for demographic variables of age and gender, the results revealed that the interaction term between TR and self-efficacy was negatively correlated with PD (*β* = −0.11, t = −3.01, *p* < 0.01). This indicated that the process by which TR related to LB through PD was moderated by self-efficacy. This indicated that self-efficacy significantly moderated the path from TR to PD (the first half of the mediation model), confirmed Hypothesis 2 (H2).

**Figure 2:**
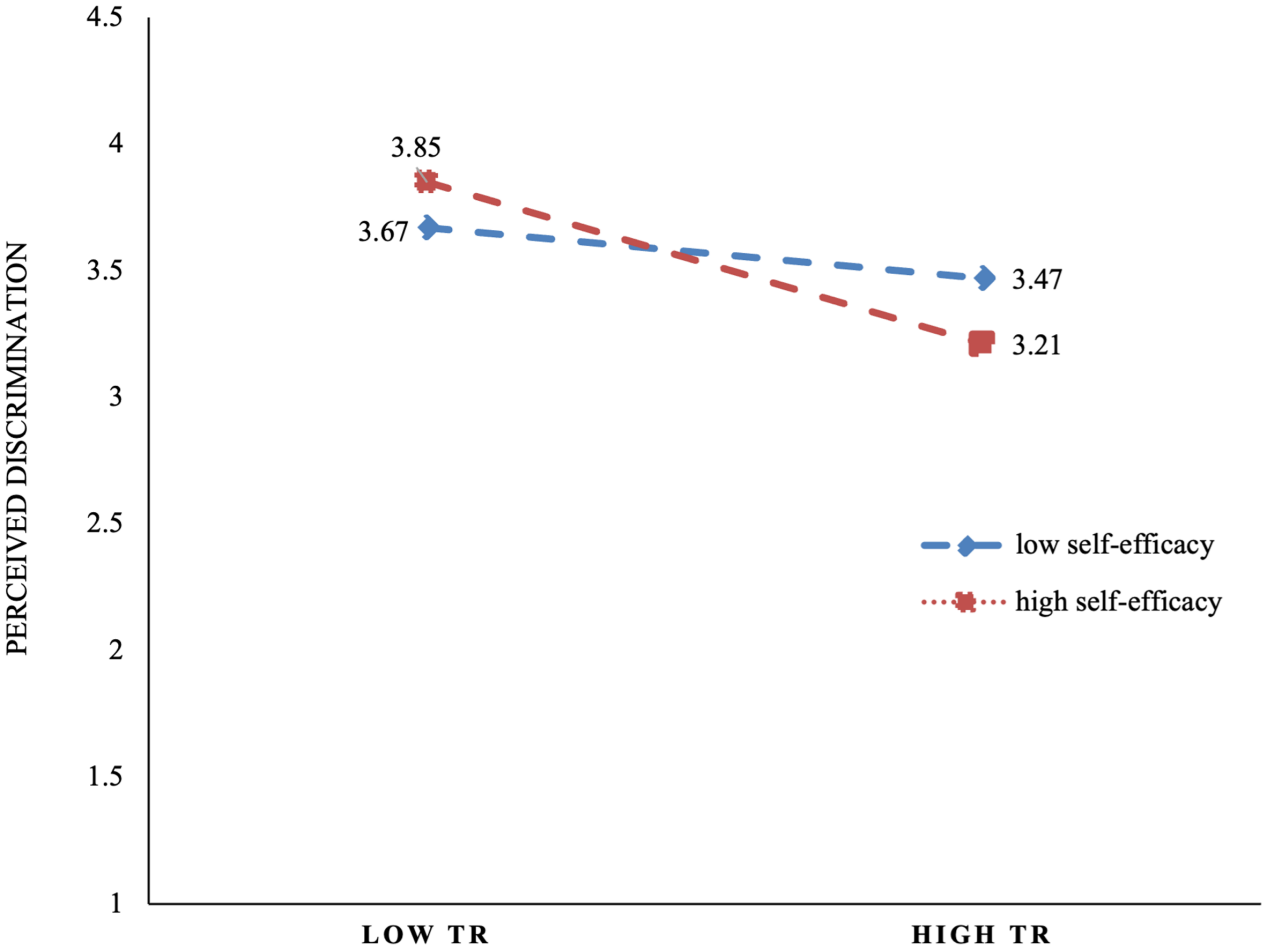
The moderating effect of self-efficacy in the relationship between TR and PD.

Simple slope analysis. To further elucidate the specific form of the moderating effect, we conducted a simple slope analysis based on high and low levels of self-efficacy (defined as one standard deviation above and below the mean, respectively; see Figure 2). For students with a lower self-efficacy level, the negative impact of TR on PD was relatively weak (b = −0.11, 95% CI = [−0.22, −0.008], *p* < 0.05); while for students with a higher self-efficacy level, the negative impact of TR on PD was stronger (b = −0.30, 95% CI = [−0.42, −0.18], *p* < 0.001). In the high self-efficacy group, the indirect effect between TR and LB through PD was relatively strong (b = 0.18, 95% CI = [−0.28, −0.08]), while in the low self-efficacy group, the indirect effect was not significant (b = −0.07, 95% CI = [−0.15, 0.003]).

## 5 Discussion

Table 2 indicated that for every one-unit increase in the TR among college students majoring in vocal music, their LB decreases by approximately 0.21 units. The findings of this study align closely with the core tenets of attachment theory in educational settings. Attachment theory posits that teachers, as significant surrogate attachment figures for students, can provide them with psychological safe havens (Bergin and Bergin, 2009). Empirical and meta-analytic studies consistently demonstrate that this high-quality emotional support from teachers and peers not only provides individuals with psychological security to cope with setbacks and prevents the decline of intrinsic motivation (Roorda et al., 2011), but also directly intervenes in individuals’ stress coping processes. As an external factor, it effectively buffers emotional exhaustion caused by high-intensity professional training and adverse environments, thereby blocking the pathway to learning burnout (Sun et al., 2025a, b).

Table 3 indicated that TR indirectly alleviates LB among college students majoring in vocal music by reducing their PD, revealing a mediating mechanism involving PD. College students majoring in vocal music frequently encounter stereotypes regarding appearance, family background, or expressive abilities. A high-attachment TR enhances students’ sense of belonging, thereby weakening the amplifying effect of these negative perceptions on burnout. Even when controlling for perceived discrimination, the direct effect c’ remains at −0.09, indicated that the protective role of the TR partially operates independently of the mediating pathway, demonstrating its robustness under multiple stressors. Overall, this mediation model provides theoretical support for psychological interventions targeting college students majoring in vocal music. Institutions can disrupt the discrimination-to-learning burnout chain by optimizing teacher-student interactions, potentially reducing LB risks in arts education while enhancing students’ overall well-being.

Table 4 shows a significant positive effect of PD on LB (*β* = 0.61, t = 21.03, *p* < 0.001). We can conclude that self-efficacy moderated the indirect effect of TR on LB mediated by PD. Specifically, as an individual’s self-efficacy increases, the mediating pathway through which the TR alleviates LB by reducing PD becomes more effective.

According to Figure 2, college students majoring in vocal music with high self-efficacy possess stronger self-confidence and a sense of control, enabling them to utilize teachers’ emotional support resources more efficiently. Consequently, as teacher-student attachment levels increase, individuals with high self-efficacy experience a greater reduction in perceived discrimination (steeper slope), and the buffering effect of the TR becomes more pronounced. For college students majoring in vocal music who have low self-efficacy, while TR still reduces pd., this reduction was relatively gradual (flatter slope) due to their lack of intrinsic efficacy to transform external support.

Based on the cross-sectional associational findings, we offer the following provisional suggestions for universities and educators regarding the alleviation of learning burnout among college vocal music majors, which await further validation through future intervention research. First, optimize the one-on-one teaching model to deepen the emotional support network between teachers and students. This study demonstrates that within the unique one-on-one teaching model of vocal education, the quality of teacher-student interaction significantly impacts students’ psychological well-being. Empirical research further indicates that positive teacher-student interactions not only fulfill students’ psychological needs for autonomy and competence but also serve as core social capital for combating academic burnout and enhancing learning enjoyment (Li and Zhang, 2024). Therefore, higher education institutions should strengthen both emotional and professional support systems, guiding faculty to establish TR oriented toward dual “skills-psychological” support. This encourages educators to proactively offer emotional understanding and guidance, thereby mitigating the intense stage performance pressures and creative burnout faced by students. Concurrently, universities can establish regular communication mechanisms between faculty and students to prevent emotional detachment from leading to student confusion and loss of academic motivation. Operationally, schools can optimize the one-on-one teaching model by implementing structured, biweekly reflective feedback sessions, allowing teachers to not only actively guide vocal skills but also focus on students’ mental health.

Second, fostering an inclusive campus culture can sever the “discrimination-learning burnout” mediating chain. This study demonstrates that perceived discrimination partially mediates the teacher-student relationship and learning burnout. Art students are particularly vulnerable to stereotypes based on appearance, family background, or communication skills in real-world settings. Eliminating implicit discrimination and workload pressures within the educational environment can effectively prevent the excessive depletion of emotional resources among music faculty and students. This is crucial for maintaining professional identity and preventing individuals from falling into academic or occupational alienation (Öztürk and Öztürk, 2020). University administrators and faculty must recognize and dismantle latent hierarchies within vocal education, establishing diverse and objective professional evaluation systems to avoid single-dimensional assessments that trigger student self-doubt and professional avoidance. By organizing non-competitive artistic exchange salons and other interactive formats, they can optimize the overall micro-ecology and break the vicious cycle of “discrimination-burnout.” Foster an inclusive campus culture by establishing objective, multi-dimensional performance evaluation rubrics and hosting non-competitive, collaborative artistic exchange salons to dismantle latent hierarchies.

Third, implement targeted self-efficacy interventions. The moderation effect analysis in this study confirms that self-efficacy significantly enhances the protective role of teacher-student attachment. Students with high self-efficacy can more effectively transform teachers’ emotional support, while those with low self-efficacy often struggle to fully leverage positive teacher-student relationships to buffer negative perceptions. Enhancing music students’ self-efficacy not only directly reduces feelings of burnout but also serves as a powerful internal resilience resource, internalizing and amplifying positive influences from the external environment (Zhu, 2025). Therefore, teachers should break down ambitious vocal learning goals into incremental, achievable sub-goals, allowing students to accumulate success experiences through phased accomplishments and steadily build their internal sense of efficacy. For students with low self-efficacy, professional evaluations should emphasize concrete improvement strategies rather than talent assessments, helping individuals build confidence and develop stronger motivation to withstand hostile external environments. In summary, to alleviate learning burnout among college students majoring in vocal music, universities must establish an intervention system where “external environmental support (high-quality teacher-student relationships and an inclusive campus culture)” and “internal psychological development (enhancing self-efficacy)” work synergistically. Only then can the practical effectiveness of educational interventions be maximized. Implement targeted self-efficacy interventions by training instructors to break down ambitious vocal learning goals into incremental, achievable sub-goals.

## 6 Limitations and future directions

Although this study explored the predicted mechanism of the teacher-student relationship on learning burnout among college students majoring in vocal music and yielded conclusions with theoretical value and practical implications, it still has the following three limitations that require further refinement in future research. First, the cross-sectional nature of the study design limits causal inference. This research employed a cross-sectional questionnaire survey method, with all data collected from the same time point. Although the research hypotheses were grounded in established theoretical frameworks and demonstrated good data fit, cross-sectional data inherently cannot establish absolute causal relationships among teacher-student relationships, perceived discrimination, self-efficacy, and learning burnout. Furthermore, while the current cross-sectional data support the proposed model, the possibility of reverse causality cannot be ruled out. For instance, students experiencing severe learning burnout might, due to their negative state, be more prone to perceiving poorer teacher-student relationships and heightened environmental discrimination. Future studies may employ longitudinal tracking designs or cross-lagged analyses to better capture the dynamic evolution of these variables across different stages of vocal training and clarify their causal directions. Second, the sample selection exhibits geographical and group limitations that affect external validity. Employing cluster sampling, the study sample comprised only 729 undergraduate vocal music students from three universities in Jilin Province, China. Given potential variations in higher education resources, campus cultures, and arts education models across Chinese regions, the representativeness of this sample is constrained. Future research should broaden the sampling scope to conduct cross-provincial and cross-institutional comparative studies. Additionally, expanding the research subjects to include other arts disciplines such as dance and fine arts could test the cross-group generalizability of this model’s conclusions. Third, data collection relied on self-reporting methods. Variables including teacher-student attachment, perceived discrimination, self-efficacy, and learning burnout were measured through student self-assessment questionnaires. Although Harman’s single-factor test results (maximum factor variance explained: 31%) indicated that the data was not severely affected by common method bias, self-assessment questionnaires inevitably remain susceptible to interference from social desirability effects or individuals’ recent subjective emotional states. Furthermore, although Harman’s single-factor test did not indicate severe common method bias, this test has inherent limitations as a diagnostic tool. Given that all variables were self-reported and collected concurrently, it remains possible that common method variance inflated the observed associations to some extent. Therefore, these findings should be interpreted with caution. Future research may explore multi-source assessment methods, such as incorporating objective evaluations of student burnout by instructors, peer nominations, or including objective indicators like course grades and performance evaluations as supplementary measures to enhance measurement objectivity and accuracy.

## 7 Conclusion

In summary, this study not only validated the mediating mechanism of “teacher-student attachment → perceived discrimination → learning burnout” but also clarified its variation across different levels of self-efficacy. This indicates that enhancing self-efficacy among college students majoring in vocal music can maximize the positive effects of teacher-student emotional support, thereby more effectively mitigating the risk of learning burnout stemming from perceived discrimination. In summary, this study not only validated the mediating mechanism of “teacher-student attachment → perceived discrimination → learning burnout” but also clarified its variation across different levels of self-efficacy. The primary novelty of this research lies in its targeted focus on the under-researched, high-pressure context of vocal music education, providing the first empirical integration of TR, PD, and self-efficacy to systematically explain and address learning burnout in arts students.

## Data Availability

The original contributions presented in the study are included in the article/supplementary material, further inquiries can be directed to the corresponding author.
